# Apoplexy, cerebrovascular disease, and stroke: Historical evolution of terms and definitions

**DOI:** 10.1590/1980-57642016dn11-040016

**Published:** 2017

**Authors:** Eliasz Engelhardt

**Affiliations:** 1 Cognitive and Behavioral Neurology Unit, INDC – CDA-IPUB – UFRJ. Rio de Janeiro RJ – Brazil.

**Keywords:** “apoplexy”, “stroke”, cerebrovascular disease, history, “apoplexia”, “acidente vascular cerebral”, doença cerebrovascular, história

## Abstract

The long-standing concept of “apoplexy' can be followed from Antiquity, passing through the Middle Ages and Renaissance, and reaching the Modern era and the present day, with the new designation of “stroke”. The definition of “apoplexy” can be divided, by the history of autopsy, into a period predating this practice, which spanned from Antiquity until the Renaissance, with a relatively stable clinically-based umbrella concept, and an autopsy period of the Modern era, when the condition was subdivided into several subtypes. Thus, it took about 2,500 years assembling the numerous pieces of information to achieve a fairly well-defined picture. The “stroke” concept inherited the information developed for “apoplexy”, incorporating all historical acquisitions to form the current state of this knowledge.

## RESUMO.

O conceito de “apoplexia”, conhecido há muito tempo, pode ser seguida desde a Antiguidade, passando pela Idade Média e a Renascença e alcançando a era Moderna e os dias presentes, com uma nova designação, “acidente vascular cerebral”. A definição de “apoplexia” pode ser dividida, pela história da autópsia, em um período precedendo essa prática, que durou da Antiguidade até a Renascença, com um conceito guarda-chuva relativamente estável baseado na clínica, e em um período de autópsia da era Moderna, quando foi alcançada a separação dessa condição em diversos subtipos. Assim, levou cerca de 2.500 anos reunindo os numerosos fragmentos de informação para chegar a um quadro satisfatoriamente bem definido. O conceito de “acidente vascular cerebral” herdou tal informação desenvolvida para a “apoplexia”, incorporando todas as aquisições históricas, para formar o presente estado de conhecimento.

## INTRODUCTION

Cerebrovascular disease refers to a group of disorders of the brain vasculature that may affect the blood supply of the underlying tissues.^1^^,^ 2 This condition should be analyzed considering the pathology of the cerebral vessels (extra- and intracranial), as well as the resultant consequences on the brain parenchyma and related structures. The diseases of the vasculature include those of the large arteries (e.g., arteriosclerosis of cerebral arteries), of the small arteries, as well as of the venous vessels, besides cardiovascular conditions (e.g., emboligenic), and systemic illnesses.^3^ The parenchymal lesions are varied, comprising ischemic changes, such as infarcts (large, small, lacunar, microinfarcts, watershed) and white matter ischemia (demyelination and axonal loss [white matter rarefaction], leukoencephalopathy), and hemorrhages (large lobar, basal ganglia, microbleeds, among others).^3^

Cerebrovascular diseases can be asymptomatic or subclinical (silent or covert), or appear as an overtly expressed clinical manifestation in the form of “stroke” (cerebrovascular accident).^1^^,^ 2 The term “stroke”, which represents an acute event leading to clinical symptoms of neural dysfunction,^4^ is regarded as having evolved from the ancient designation “apoplexy”, which likewise refers to a clinical concept characterized by rapid loss of consciousness, and various manifestations of brain dysfunction. The “apoplexy” concept used to embrace varied disorders, later identified as acute cerebral events, vascular and non-vascular (e.g., abscess, hydatids, pus, tumors, among others), as well as non-cerebral acute occurrences (e.g., myocardial infarction, pulmonary embolism, intoxications, among others).^5^^,^ 6 The term “stroke” was introduced in the course of the historical studies and after a long time came to replace “apoplexy”, a term which has virtually disappeared from the medical literature.^7^

The historical highlights of the above-mentioned terms and concepts will be outlined, with a focus mainly on Western medicine, from Antiquity to the Modern era.

## APOPLEXY: TERM AND CONCEPT

The term (*apoplexia*) (“struck down with violence”, “to strike suddenly”) has been used since Antiquity, identifying, in the ancient and clinical sense of the term, a disorder in which “a person suddenly falls, without consciousness or motion, retaining pulse and respiration”.^5^^,^ 6 ^,^ 8 This characteristic picture was well known to the ancient Greeks, at least 2,500 years ago, and “in all probability, long before, it was mentioned in writings that have not come down to us, and by authors whose names have been entirely forgotten”.^9^

## ANTIQUITY: THE GRECO-ROMAN PERIOD

This period spans about 1,000 years (c. 500 BC-c. 500 AD), when the bases of the concept was established. Many authors contributed with their knowledge, and despite lost documents, a coherent narrative took shape. Some of the many authors will be named.^10^

Hippocrates (and the Hippocratic Corpus) is responsible for the first recorded appearance of the term “apoplexy”. The concept is mentioned in several parts of his extensive work, describing the clinical picture, with some variations^5^^,^ 11 (Box 1).

**Box 1:** Definition of "apoplexy" in the Greco-Roman era.

**Hippocrates.** "Apoplexy" definition (not explicit) (De Morbis II): "Suddenly a healthy person is seized with head pain, immediately the voice fails, he snores, and the mouth is open (gapes), and if someone calls or moves, he only groans, nothing with meaning, gives (releases) copious urine, and does not perceive. If the fever does not seizes (appears), he dies in seven days. Because it seizes (comes), the health is generally spared".5 And: "Suddenly seized with pain to the head, immediately the voice fails, and he becomes incapacitated (powerless). Here, within seven days, unless the fever seizes, he dies. If the attack is strong".11
"Apoplexy" definition (explicit) (Aphorisms II-42): "It is impossible to remove a strong attack of apoplexy, and not easy to remove a weak attack".^14^
**Galen.** Definition (De loci affectis, Commentary on Hippocratic Aphorisms): "Apoplexy is an unconsciousness of the mind, with a privation of the senses and a palsy of the body." Also: "Apoplexy is a privation of sense and motion in all the nerves". And: "In apoplexy the whole body suddenly is deprived of sensation and motion, with only respiration remaining, and if it is prevented, the apoplexy is maximum and very severe".12,13

The Hippocratic age was followed by numerous personalities, such as Celsus (c. 25 BC-c. 50 AD), Aretaeus (c. I century AD), Archigenes (c. I-II century AD), Galen (129 to c. 210 AD), Caelius Aurelianus (c. IV-V century AD), among many others.^10^ Galen deserves a special mention. He acknowledged the current notions on the theme, which he developed with ideas somewhat divergent from those of Hippocratic authors, maintaining, however, the original core^12^^,^ 13 (Box 1).

It is important to remind that, at the time, the “humoral theory” (humors, spirits), as well as “divine punishment”, was plainly in vogue as a determining factor of the ailment, a notion that would begin to be abandoned only in the 18^th^ century of the Modern era.^7^^,^ 10 ^,^ 13

## MIDDLE AGES OR MEDIEVAL ERA

The Medieval history of apoplexy, extending from the 5^th^ to the 14^th^ century, was represented by numerous prominent names. The authors of the early Middle Ages maintained the Hippocratic-Galenic ideas, with minor variations. The high Middle Ages included Byzantine, Persian, and Arabic authors, and in the high and late epoch the authors were from Western Europe, mainly Italy, France, and Germany. The entire period was influenced by ideas from Greco-Roman and Arabic works, but without the introduction of novel concepts.^16^^-^ 18

## RENAISSANCE

The Renaissance, between the 14^th^ and 17^th^ century (in Europe), witnessed a renewed rise of interest in the ancient Greco-Roman ideas. At the time, translations of the ancient works were performed. The promotion of human dissection (autopsy) allowed society to use this for forensic, health and scientific purposes, and led to the emergence of new knowledge. The discoveries during the medical Renaissance paved the way for modern medicine.^19^

During the Greco-Roman, Medieval and Renaissance periods the term “apoplexy” was maintained by all authors, along with its umbrella meaning. Important changes would come with the autopsy findings of the ensuing period.

## MODERN ERA

Many changes occurred from the 17^th^ century on with the advent of autopsies, and “apoplexy” began to lose its unitary (umbrella) meaning.^20^ The non-vascular intracranial and the systemic conditions began to be separated, and only the vascular-related events retained the original designation. A classification started to emerge, based on cadaveric examination, dividing the condition into increasingly numerous subtypes, and laying the foundations of the study of the chapter of “cerebrovascular disease(s)”. Some of the most cited authors of the period,^21^^,^ 22 and their contributions to the theme, will be mentioned, without diminishing the influence of many others (Box 2).

**Box 2:** "Apoplexy" in the Modern era (from 17th century on): discovery of subtypes timeline – authors and excerpts.

**Johann Jakob Wepfer** (1620-1695) (Swiss). Distinguished for the first time non-traumatic intracranial bleeding - in one case: "...cut the dura mater, much blood discharged from the space between it and the pia-mater [subarachnoid space] ..."; in another case: bleeding with ruptured anterior cerebral artery branch; in one more case, with serous liquid (pale-colored serum) accumulation (1658).^23^
The (non-traumatic, spontaneous) "hemorrhagic apoplexy" [intracranial hemorrhage], [subarachnoid hemorrhage], and [cerebral hemorrhage] due to vascular cause, and a non-hemorrhagic variety, emerged.
**William Cole** (1635-1716) (English). The first to use the term "stroke" to denote "apoplexy" in English medical writing (1689).^24^
**Franciscus (Francesco) Biumi** (xxxx-xxxx) (Italian). Described a case of "apoplexy", without intracranial bleeding, identifying: "...internal carotid aneurysmal sack [saccular aneurysm] in the cavernous sinus (Vieussens receptacle)..." [aneurysm - unruptured] (1765).^25^
**Giovanni Battista Morgagni** (1682-1771) (Italian). Distinguished a "sanguineous" (with bleeding) [hemorrhagic], a "serous" (with intracranial fluid) [non-hemorrhagic], and a "neither sanguineous nor serous" type apoplexy (1761).^26^
**John Blackall** (1771-1860) (English). Described: "...haemorrhage in the space between the meninges... [subarachnoid hemorrhage] ...traced to the basilar artery...at its bifurcation was dilated into an aneurysmal sac [basilar artery aneurysm] ... opened into the cavities" [aneurysm - ruptured] (1814).^27^
**Jean André Rochoux** (1787-1852) (French). Defined "hemorrhagic apoplexy" [cerebral hemorrhage], and introduced the term *ramollissement du cerveau* [softening of the brain ] [infarction] (1814).^28^
**Léon Louis Rostan** (1796-1866) (French). Defined "sanguine (hemorrhagic) apoplexy" [cerebral hemorrhage] and extended the notion of (noninflammatory) "cerebral softening" (*ramollissement cérébral*) [cerebral infarction] (1819); he considered "apoplexy" only for hemorrhagic events.^29^
**Améedée Dechambre** (1812-1886) (French). Introduced the term *lacune* [lacune] for small rounded cavities probably resulting from the liquefaction after partial resorption within foci of softening (1838).^30^
**Charles Louis Maxime Durand-Fardel** (1815-1899) (French). Described "hemorrhage" (meningeal, cerebral) [subarachnoid hemorrhage] [cerebral hemorrhage], "brain softening" [cerebral infarction], "interstitial atrophy of the brain" [white matter rarefaction (demyelination and axonal loss)], "lacune", and *état criblé du cerveau* (riddled state of the brain).^31^
**Rudolf Ludwig Karl Virchow** (1821-1902) (German). Introduced the terms apoplexia sanguinea (*Hämorrhagische Apoplexie des Gehirns*) (hemorrhagic apoplexy of the brain) [cerebral hemorrhage], and apoplexia ischaemica (ischaemic apoplexy) (*Hirnerweichung*) [cerebral softening] [cerebral infarct], caused by embolism; created the terms "thrombosis" and "embolism"; revived the term "arteriosclerosis" (according to Lobstein, 1829) (1852).^32^
**Jean-Baptiste Vincent Laborde** (1830-1903) (French). Described the "pisiform lacunes" (*lacunes pisiformes*) as "...small cystic cavities...small blood effusions that have suffered a complete resorption...in other cases ...their centers ...resulting from partial and progressive disorganization..." for "lacunes" [post-hemorrhage and post-softening] (1866).^33^
**Julius Friedrich Cohnheim** (1839-1884) (German). Introduced the terms *Infarct* [infarct, infarction], *Nekrose* [necrosis] and *hämorraghischer Infarct* [hemorrhagic infarct], after embolic obstruction of terminal arteries (1872).^34^
**Otto Ludwig Binswanger** (1852-1929) (Swiss). Described marked atrophy of the subcortical white matter he named "Chronic progressive subcortical encephalitis" (1894), later named after him, and subsequently known as "Binswanger disease".^35^^,^ 36
**Alois (Aloysius) Alzheimer** (1864-1915) (German). Described diffuse and focal subtypes of vascular injuries of the brain, including focal cortical atrophic changes (*senile Sklerose der Hirnrinde and senile Rindenverödung*), "cortical microinfarcts" (1899-1902).^37^^,^ 38

Wepfer (1658) identified non-traumatic intracranial hemorrhage – “subarachnoid hemorrhage” and “cerebral hemorrhage”, as separate from other “apoplexy” types.^23^

Wepfer split, for the first time, the unitary “apoplexy” definition, marking the start of studies on vascular diseases of the brain or “cerebrovascular disease(s)”.

- Cole (1689) first used the term “stroke” to denote “apoplexy” in English medical writing .^24^
- The term would be adopted much later.
- Biumi (1765) described a case of a non-ruptured saccular “aneurysm” of the carotid artery.^25^
- Morgagni (1761) distinguished a “sanguineous” [intracranial hemorrhage], a “serous” [non-hemorrhagic], and a “neither sanguineous nor serous” apoplexy.^26^
- Blackall (1814) identified a ruptured “aneurysm” of the basilar artery in a case of “subarachnoid hemorrhage”.^27^
- Rochoux (1814) defined a “hemorrhagic apoplexy” [cerebral hemorrhage] and introduced the term *ramollissement du cerveau* [softening of the brain] [infarction].^28^
- Rostan (1819) defined “sanguine (hemorrhagic) apoplexy” [cerebral hemorrhage] and extended the notion of non-inflammatory *ramollissemente cérébral* [cerebral softening] [cerebral infarction].^29^

Here, the basic distinction between the two main forms of “apoplexy”, hemorrhagic and ischemic (“cerebral hemorrhage” and “cerebral infarction”) became clearly recognized. This division was set to endure until the present day, maintained also for the “stroke” concept that would follow.

- Dechambre (1838) introduced the term *lacune* [lacune].^30^
- Durand-Fardel (1843) described “hemorrhage” (meningeal, cerebral) [subarachnoid hemorrhage] [cerebral hemorrhage], “brain softening” [brain infarction, cerebral infarction], “interstitial atrophy of the brain” [white matter rarefaction], “lacune” and état *criblé du cerveau* [riddled state of the brain].^31^
- Virchow (1852) introduced the terms *apoplexia sanguinea* [cerebral hemorrhage] and embolic *apoplexia ischaemica* (ischaemic apoplexy) [cerebral softening, cerebral infarct], created the terms “thrombosis” and “embolism”, and revived the notion of “arteriosclerosis”.^32^
- Laborde (1866) described *lacunes pisiformes* [“lacunes”], post-hemorrhagic and post-softening.^33^
- Cohnheim (1872) introduced the terms “infarct”, “necrosis” and “hemorrhagic infarct”, following embolic obstruction of terminal arteries.^34^
- Binswanger (1894) described “Chronic progressive subcortical encephalitis” (later named after him, and subsequently known as “Binswanger disease”).^35^
- Alzheimer (1898) described “cortical microinfarcts”.^37^

At this time, the main subtypes of “apoplexy” due to “cerebrovascular disease(s)” were identified, and would, as a next step, be officially recognized and reunited in a reference report.

## STROKE AND ILCD/ICD: PRESENT-DAY TERMS AND DEFINITIONS

The term “apoplexy” was maintained as a medical term in the 4^th^ Revision of the International List of Causes of Death (ILCD-4) (1929). The term was abandoned from the 5^th^ Revision onwards (ILCD-5) (1938).^39^

The term “stroke” (cerebrovascular) appeared first in the ICD-9 (1968).^39^ The definition of the concept by the World Health Organization (WHO) appeared soon after (1971 and 1980),^4^^,^ 39 and more recently, a new definition was proposed by the American Heart Association-American Stroke Association (AHA-ASA) (2013)^15^ (Box 3).

**Box 3:** Definitions of "stroke" in the Modern era (20^th^-21^st^ century) (excerpts as exemples).

**WHO (World Health Organization)**
• "Stroke" definition (1971): "A sudden onset of disturbance of focal bran function due to the blockage or rupture of blood vessels."^40^
• "Stroke" definition (1980): "Rapidly developing clinical signs of focal (or global) disturbance of cerebral function, lasting more than 24 hours or leading to death, with no apparent cause other than that of vascular origin."^4^
**AHA-ASA (American Heart Association-American Stroke Association) (2013)** 15
• Ischemic "stroke": "An episode of neurological dysfunction caused by focal cerebral, spinal, or retinal infarction."
• Intracerebral hemorrhagic "stroke": "Rapidly developing clinical signs of neurological dysfunction attributable to a focal collection of blood within the brain parenchyma or ventricular system that is not caused by trauma."
• Subarachnoid hemorrhagic "stroke": "Rapidly developing signs of neurological dysfunction and/or headache because of bleeding into the subarachnoid space, which is not caused by trauma."
• "Stroke" (not otherwise specified): "An episode of acute neurological dysfunction presumed to be caused by ischemia or hemorrhage, persisting ≥ 24 hours or until death, but without sufficient evidence to be classified as one of the above."

The notion “cerebrovascular disease(s)” was introduced initially as “arteriosclerosis with cerebral vascular lesion” in the ILCD -3 (1920), and replaced by “cerebrovascular disease(s)” from the ICD-8 (1965) onwards.^39^

The current ICD-10 (1990) (version 2016), contains the term “cerebrovascular disease(s)” and “stroke” (cerebrovascular accident), besides “intracerebral hemorrhage”, “subarachnoid hemorrhage”, “cerebral infarction” (thrombosis, embolism), “lacunar” (syndromes), “Binswanger disease”, “cerebral aneurysm”, “subarachnoid hemorrhage”, among others.^39^

Thus, after an extensive period of research, the WHO went on to incorporate progressively, in the official reference report, all subtypes of “cerebrovascular disease(s)” that were so arduously developed. However, the term “apoplexy”, with a more restricted meaning, remains in use in the medical literature to the present day.

## CONCLUSION

“Apoplexy” is a long-known condition. Since Hippo­crates, or even earlier, numerous authors dedicated their talents to studying this subject. In the days before autopsies were allowed, from Antiquity until the Renaissance, the definition was relatively stable, an all-embracing concept for a broad condition. The advent of autopsies in the Modern era allowed the concept to be further refined and broken down, and several subtypes emerged, furthering understanding on the subject. “Stroke” inherited the information that was developed for “apoplexy”, incorporating all historical acquisitions, to form the current state of this knowledge.
